# The Cross‐Cultural Interplay of Visual Attention and Artistic Design in Comics: Insights From Eye‐Tracking Evidence on American and Japanese Readers

**DOI:** 10.1111/cogs.70091

**Published:** 2025-07-22

**Authors:** Yuki Shimizu, Motohiro Kozawa, Keiichi Watanuki, James S. Uleman, Honami Arihara

**Affiliations:** ^1^ Faculty of Letters, Arts, and Sciences Waseda University; ^2^ Graduate School of Education Saitama University; ^3^ Graduate School of Science and Engineering Saitama University; ^4^ Department of Psychology New York University; ^5^ Faculty of Human Sciences Mejiro University

**Keywords:** Artistic design, Comic reading, Cross‐cultural, Visual attention, Visual narrative

## Abstract

This study investigated cross‐cultural differences in visual attention patterns during comic reading, focusing on participants with Japanese and American cultural backgrounds. Using an eye‐tracking paradigm, we examined attention processes as participants viewed pages from American comics and Japanese manga featuring objective or subjective viewpoints. The results showed that for objective pages, American readers exhibited relatively longer fixations on focal objects, while Japanese readers allocated relatively more attention to backgrounds, aligning with analytic versus holistic cognitive styles. By contrast, for subjective materials, Japanese readers demonstrated greater attention to focal objects than American readers did, suggesting that the subjective perspective embedded in manga shifts Japanese readers toward a focal‐object‐oriented attentional style. Individual differences in self‐reported analytic‐holistic cognitive styles and manga reading experience, in addition to cultural background, were associated with attentional patterns for manga. The results underscore the influence of artistic design in shaping visual attention in ways that both mirror and transcend culturally ingrained attentional biases. This study deepens our understanding of cross‐cultural variations in visual processing and comic reading behaviors, providing fresh insights into the complex interplay among culture, cognition, and visual narrative comprehension.

## 1 Introduction

Culture influences people's cognitive functions, such as attention (Markus & Kitayama, 1991). Overt visual attention is guided by bottom‐up (e.g., stimulus salience) and top‐down (e.g., task demands, knowledge, and goals) processes (Carrasco, 2011; Parkhurst, Law, & Niebur, 2002; Pearson, Watson, Cheng, & Le Pelley, 2020). Culture consists of shared meanings and concepts (Shweder et al., 2006), and it shapes people's attention mainly through top‐down processes (Kitayama & Salvador, 2017; Markus & Kitayama, 1991; Nisbett, Peng, Choi, & Norenzayan, 2001). Cross‐cultural studies on visual processing have indicated systematic cultural variations when viewing scenes that contain focal objects within a background (Chua, Boland, & Nisbett, 2005; Masuda & Nisbett, 2001; Miyamoto, Nisbett, & Masuda, 2006). For example, when asked to describe short animated vignettes, participants from Japan were more likely to mention the context of objects and the relationships among them, whereas participants from the United States were more likely to focus on objects (Masuda & Nisbett, 2001). This cultural difference was replicated in an eye‐tracking study: North Americans looked at focal objects sooner and longer than Asian participants, who were more likely to fixate their eyes on the background (Chua et al., 2005).

This empirical evidence provides support for the theoretical framework of analytic versus holistic cognitive styles proposed by Nisbett and his colleagues (Nisbett, 2003; Nisbett & Miyamoto, 2005; Nisbett et al., 2001). Analytic cognition, which is predominant in Western cultures, is characterized by a focus on objects and their attributes independent of context. This style emphasizes the use of formal rules and logic to categorize objects and resolve contradictions, reflecting an orientation toward objectivity. In contrast, holistic cognition, which is more commonly observed in East Asian cultures, emphasizes the relationships between objects and their broader context. This cognitive style is marked by attention to situational and relational factors, and a preference for thematic rather than categorical classification.

Culturally dominant attention patterns are manifested in artwork production, such as picture drawings (Masuda, Gonzalez, Kwan, & Nisbett, 2008; Nand, Masuda, Senzaki, & Ishii, 2014; Senzaki, Masuda, & Nand, 2014). Historically, East Asian landscape pictures have tended to employ a bird's‐eye perspective, including more context, whereas Western landscape pictures tend to employ a linear perspective, focusing on foreground objects. The analysis of 15–19th century Western and East Asian paintings demonstrated that traditional East Asian artworks are more likely than traditional Western artworks to emphasize contextual elements, such as placing the horizon higher, including more pieces of information, and drawing people smaller (Masuda et al., 2008). Such culture‐specific characteristics are also present in contemporary populations (Nand et al., 2014; Senzaki et al., 2014).

However, these cultural differences are not observed in all categories of artistic expression. Comics, which are visual narratives that combine images and texts in sequential panels and are classified as artwork (Ball, 2020), exhibit unique cultural variations. Studies comparing panel compositions in American comics and Japanese manga (hereafter manga) have uncovered notable distinctions in visual narrative techniques (Cohn, 2024; Cohn, Hacımusaoğlu, & Klomberg, 2023; Cohn, Taylor‐Weiner, & Grossman, 2012). American comics predominantly utilize an *objective* viewpoint, employing more macro panels that depict entire scenes, thereby allowing readers to observe a scene in its entirety from a distant perspective. In contrast, manga frequently adopts a *subjective* viewpoint, emphasizing individual elements, including characters and specific scene components. This latter approach engenders a sense of personal immersion, as readers perceive the narrative through their own viewpoint within the story.

This divergent viewpoint pattern in comics has produced the following speculations. The cross‐cultural differences in viewpoints within comic panels reflect comic artists’ simulations of readers’ attentional processes: manga artists may simulate Japanese readers’ tendency to allocate their attention to the whole scene and, therefore, employ subjective viewpoints attempting to direct readers’ attention to focal objects or characters. Conversely, American comic artists may assume that American readers tend to focus on focal objects or characters independently, thus allowing the employment of objective panels (Cohn, 2024; Cohn et al., 2012).

Nevertheless, to the best of our knowledge, no empirical research has examined this interaction between cultural variations in the artistic design of comics and readers’ cultural backgrounds. Moreover, no empirical study has investigated the attentional processes involved in reading diverse types of comic books cross‐culturally. Several studies have investigated participants’ eye movements in reading comics using eye‐tracking (Foulsham & Cohn, 2021; Foulsham, Wybrow, & Cohn, 2016) but have not employed a cross‐cultural approach. Therefore, the current study empirically examined the interaction between readers’ cultural backgrounds and comic materials on visual attention processes during comic reading. The specific objective was to determine whether participants from Japan and the United States exhibit different patterns in allocating attention to focal objects and backgrounds when reading comic pages from subjective or objective viewpoints. This approach allows us to integrate findings from cross‐cultural, visual attention, and comics studies.

In previous studies examining the processes of viewing comics, panels have frequently been used as stimuli. However, in this study, actual comic pages were employed as stimuli, for several reasons. First, in comics, panels are arranged according to the artist's intentions within the page (Cohn, 2024), and certain artistic techniques, such as elements that extend beyond panel boundaries or full‐page splash illustrations, contribute to the complexity of comic layouts. Therefore, the page layout can be interpreted as reflecting the comic artist's intentions, which enables us to investigate the interaction between the material and the reader. Second, by adopting pages as the unit of analysis, we can observe comic reading behavior in a manner that closely approximates the authentic reading experience.

As previously noted, American comics tend to employ an objective viewpoint, whereas manga tends to employ a subjective viewpoint (Cohn et al., 2023; Cohn et al., 2012). However, given that this study included participants from both Japan and the United States, it is anticipated that their familiarity with each comic type will influence their attention processes. Therefore, this study extracted pages from American comics and manga that primarily utilized either an objective or a subjective viewpoint, employing these as stimuli. This methodology facilitates a direct examination of the interplay between the viewpoints utilized in materials and readers’ cultural backgrounds.

Based on extant empirical evidence of cross‐cultural differences in viewing visual scenes (Masuda & Nisbett, 2001; Miyamoto et al., 2006) and cultural variations in comic materials (Cohn et al., 2023; Cohn et al., 2012), we hypothesized that more culturally characteristic attention patterns would be observed in stimuli from an objective viewpoint, wherein the focus of gaze on focal objects is not guided. Specifically, it was predicted that, when viewing objective pages, the American group would exhibit a greater focus on focal objects than the Japanese group, while the Japanese group would demonstrate increased attention to background information relative to the American group.

Furthermore, this study examined whether individual differences in analytic‐holistic cognitive styles and comic reading experiences can serve as potential predictors of visual attention during comic reading. We hypothesized that individuals with a more holistic attention style would exhibit a reduced attentional focus on focal objects when viewing objective pages. Furthermore, it was predicted that individuals with greater experience reading comics would demonstrate increased attentional focus on focal objects, even when reading objective pages.

## 2 Method

### 2.1 Participants

A total of 64 undergraduate students participated in this study, including 30 students from the United States (13 men, 16 women, and 1 other, *M*_age_ = 20.90, *SD*_age_ = 2.67) and 34 students from Japan (15 men and 19 women, *M*_age_ = 20.15, *SD*_age_ = 1.91). An additional 16 participants were excluded from data analyses because of insufficient looking time (less than 70% of the demonstration; six American and five Japanese) or failure of the eye‐tracker (three American and two Japanese). A power analysis using G*Power (Faul, Erdfelder, Lang, & Buchner, 2007) revealed that 44 participants were required to detect a medium effect size (*f* = 0.25) with sufficient power (1 – β = 0.95). Therefore, the sample size of this study was considered sufficient. American participants were recruited from a subject pool at New York University in the United States, and Japanese participants were recruited from an introductory psychology class at Saitama University in Japan. The ethnic makeup of the American participants was as follows: 10 White Americans, seven Asian Americans, six Hispanic Americans, four African Americans, and three mixed races. All participants from Japan were ethnically Japanese. The first language of all American participants was English, and that of all Japanese participants was Japanese. The American participants received course credit, and the Japanese participants received 1000 Japanese yen (approximately eight dollars) for their participation. Written informed consent was obtained from all participants. All procedures involving human subjects in this study were approved by the ethics committees of New York University and Saitama University, where the data collection was conducted. All measures, manipulations, and data exclusion for the experiments reported here are disclosed. Data collection was not continued after the data analysis.

### 2.2 Stimuli

The stimuli comprised 40 comic pages extracted from existing American comics and manga, which were available in both Japanese and English (see Appendix A for a list of sources for the stimulus pages). For each set of 20 American comics pages and 20 manga pages, half (10 pages) comprised subjective pages, and the other half (10 pages) consisted of objective pages. Subjective pages typically used close‐up views, point‐of‐view perspectives, and rapid shifts in focal characters, encouraging immersion into the character's experience. In contrast, objective pages featured wider shots with multiple characters and detailed backgrounds, presenting scenes from a neutral observer's perspective. The Japanese and English versions of the stimuli were identical except for the language of the text. The average number of panels included per page was 4.8 for American comics and 5.5 for manga. The pages are discrete and do not form a continuous sequence. Noncontinuous pages were selected to allow precise experimental control over the manipulation of viewpoint (subjective vs. objective). Using sequential pages would have introduced narrative and perspectival variability that could confound the viewpoint manipulation. By employing standalone pages validated through pretesting, we minimized confounding factors and aimed to isolate differences in visual attention primarily tied to viewpoint framing.

The stimuli were selected and validated through a pretest with 16 American and 18 Japanese participants. After being provided with definitions of each viewpoint, the pretest participants rated 60 pages on a 7‐point scale (1 = objective, 7 = subjective). Pages scoring above 4.5 were categorized as subjective, and those below 3.5 as objective. Full results are presented in Appendix B. While our classification relied on participant‐based ratings of subjectivity, we acknowledge that alternative frameworks—such as those proposed by Cohn (2024) and Atilla, Klomberg, Cardoso, and Cohn (2023)—define subjectivity based on formal visual features, including panel composition, point‐of‐view shifts, and the degree of background elaboration. Whereas these structure‐based approaches provide a principled account of narrative design conventions, our method captures the subjective impressions of viewers, thereby offering a complementary perspective on how such features are psychologically experienced during comic reading.

### 2.3 Procedure

Participants were tested individually in an experimental room. Before the commencement of all sessions, participants were informed that this study would investigate how individuals read comics. Instructions for all tasks were presented in the participants’ native language. The experiment consisted of two sessions: a comic viewing session and a questionnaire session.

In the *Comic Viewing session*, participants were instructed that they would be shown various pages of comics and that each page would appear separately and independently. The participants were presented with comic stimuli page‐by‐page on the display of a 17‐inch laptop computer, with an interval page showing a cross in the center inserted for 1 s between pages. The presentation order was counterbalanced, with half of the participants viewing the 20‐page American comics first, and the other half viewing the 20‐page manga first. Before reading American comics, the participants were instructed to read from left‐to‐right and top‐to‐bottom, and before reading manga, they were instructed to read from right‐to‐left and top‐to‐bottom. The participants were instructed to view each page at their own pace and press a key to proceed to the next page. The order of page presentations was randomized across the participants.

The participants sat on a chair approximately 65 cm from the monitor. The monitor size was 38 cm × 21 cm (width × height), and the stimulus pages were displayed at approximately 12.5 cm × 18.5 cm, subtending a visual angle of 10.98° × 16.20° when viewed from a distance of 65 cm. This ensured that the stimuli appeared at a size comparable to typical printed comic pages. The participants’ eye movements for each presented page were measured using a Tobii Pro X3‐120 (Tobii Technology, Sweden) at a sample rate of 120 Hz and a resolution of 1920 × 1080 pixels. Before presenting the comic pages, a 5‐point calibration was completed using Tobii Studio software. While viewing each page, the participants were instructed to indicate their level of interest in each material by manipulating a lever. However, interest‐level data were excluded from the analyses owing to insufficient documentation of participants’ lever operations.

In the *Questionnaire session*, participants were first asked to complete the 24‐item Analysis‐Holism Scale (AHS) (Choi, Koo, & Jong An, 2007) on a 7‐point scale (1 = *strongly disagree* to 7 = *strongly agree*). A higher score indicated a more holistic style, whereas a lower score indicated a more analytical style. Then, the participants answered the questions regarding their experience of reading comics (“How often do you currently read manga [American comics]?”) using a 7‐point scale (1: “I have never read manga [American comics],” 2: “I used to read manga [American comics] but not anymore,” 3: “Several times a year,” 4: “Once a month,” 5: “Once a week,” 6: “More than twice a week,” and 7: “Everyday”).

### 2.4 Data analyses

**Fig. 1:**
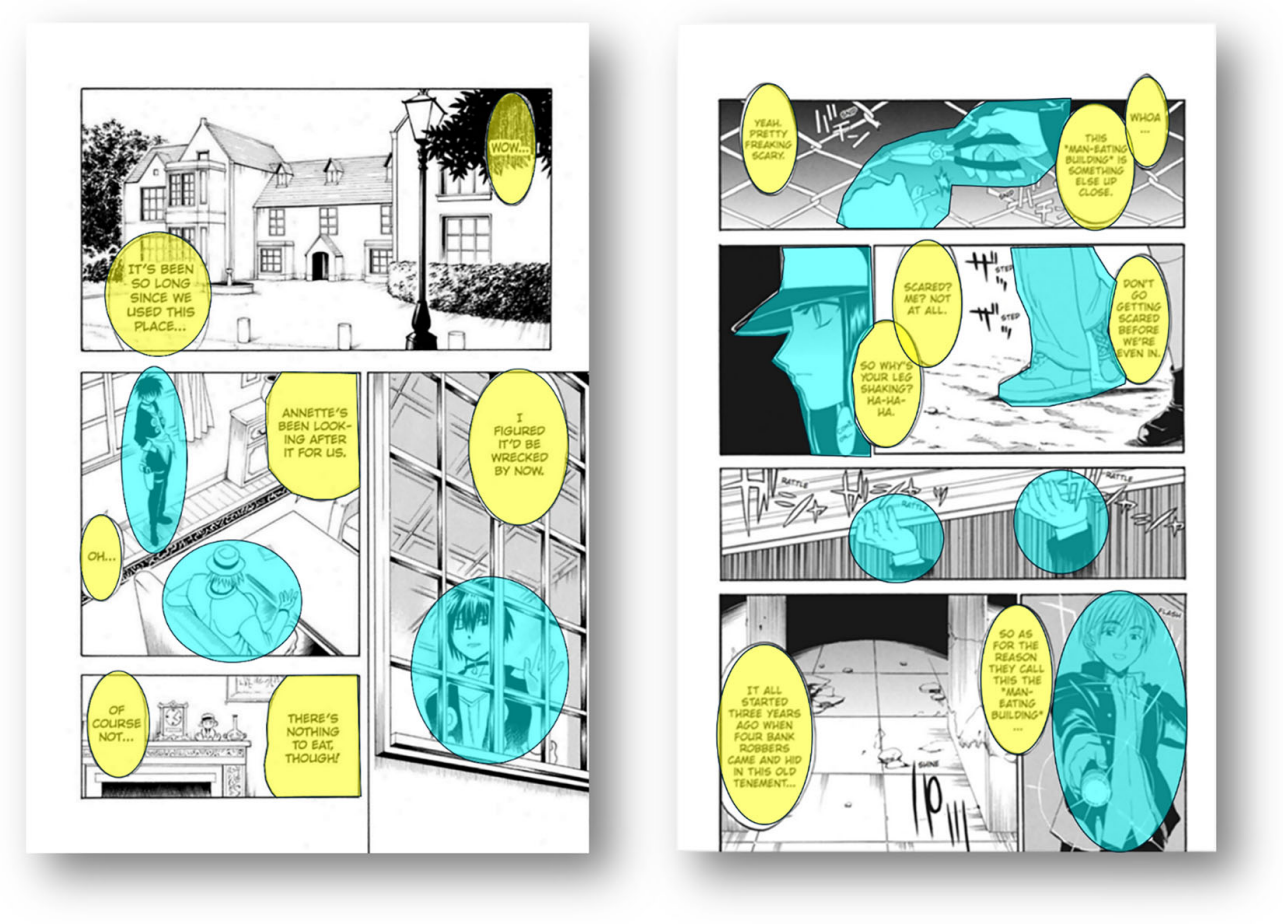
Examples of Areas of Interest (AOIs) in objective (left) and subjective (right) pages. *Note*. The blue regions indicate the Areas of Interest (AOIs) of the *focal object*, whereas the yellow regions indicate the AOIs of the *balloon*. All areas not included in the focal object and balloon were categorized as the *background*. The source of the left image is “BLACK CAT” by Kentaro Yabuki, SHUEISYA, vol. 1, p. 104, and that of the right image is “Corpse Princess” by Yoshiichi Akahito, Square Enix, vol. 1, p. 23.

Areas of interest (AOIs) were created on each page, comprising the focal object, balloon, and background. The *Focal object* included characters or objects that corresponded to at least one of the following: (1) the person speaking a line; (2) the central person in the interaction; or (3) the person or object occupying most of the frame. The *balloon* included not only a normal balloon with dialogue but also text not enclosed by a balloon, such as situation explanations. Two experts, an artist and a psychologist (both included in the authors), independently decided where to draw the AOIs. The agreement rate between the two judges was 93.5%. Disagreements were resolved by discussion. On each page, all areas not included in the focal object and balloon were categorized as *background*. Therefore, each page was divided into three areas: the focal object, balloon, and background (see Fig. 1 for examples of drawing AOIs). The average area proportions of focal object and balloon AOIs (relative to total page area) were as follows: American comics: focal object = 22.33% (objective), 33.97% (subjective); balloon = 10.62% (objective), 5.41% (subjective); manga: focal object = 17.04% (objective), 31.68% (subjective); balloon = 15.22% (objective), 10.10% (subjective).

For each participant, the proportion of the fixation duration for each AOI relative to the total fixation duration on each page was calculated. The *Shapiro−Wilk* test of normality suggested that not all proportion scores were normally distributed; therefore, the scores were arcsine‐transformed before the analyses. To explore cultural differences in eye‐tracking variables, arcsined proportion scores were analyzed using a 2 (Culture: American, Japanese) × 2 (Medium: American comics, manga)× 2 (View: subjective, objective) × 3 (AOI: focal, balloon, background) four‐way mixed‐design analysis of variance (ANOVA), with Culture as a between‐participants variable and Medium, View, and AOI as within‐participants variables. All tables and figures present the original and untransformed data. Hierarchical regression analysis was conducted to investigate the effects of each participant's analytic–holistic cognitive style and comic reading experience on attentional bias. We also conducted supplementary regression analyses to examine whether the subjectivity ratings obtained from a pretest for each comic page predicted participants’ visual attention. Preliminary analysis of gender and task order revealed no significant effects for any measure; therefore, all analyses collapsed across gender and task order.

## 3 Results

### 3.1 Visual attention to subjective and objective pages

**Fig. 2:**
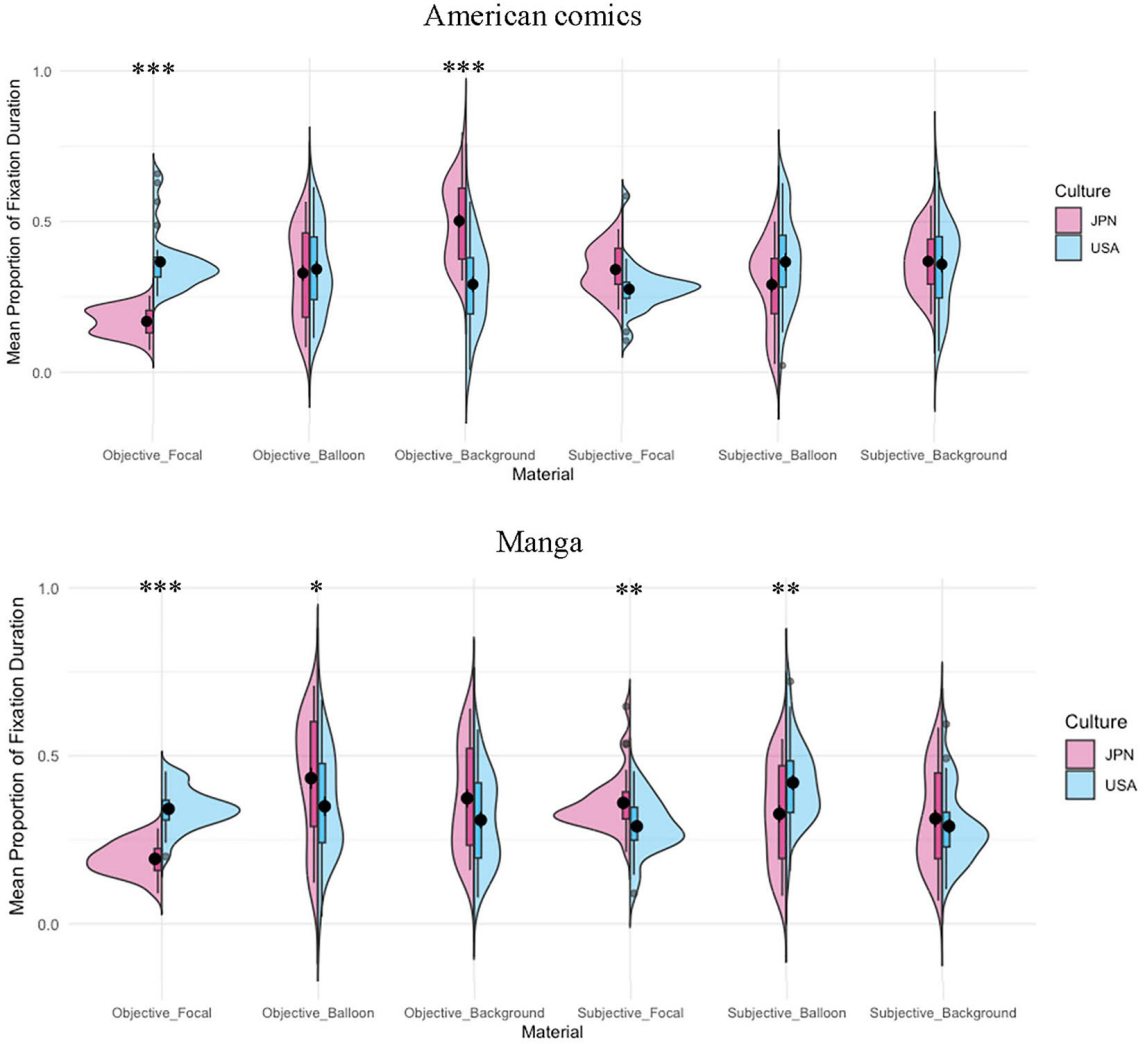
The mean proportions of the fixation durations for each AOI relative to the total fixation duration for each page. *Note*. The black dots indicate the mean value, and the error bars indicate standard errors. ***p < .001; **p < .01; *p < .05.

The mean proportion of the fixation duration on each AOI (focal object, balloon, and background) relative to the total fixation duration for each page was calculated and averaged for each type of material, as presented in Table 1. A 2 (Culture) × 2 (Medium) × 2 (View) × 3 (AOI) ANOVA revealed a four‐way interaction, *F*(1.53, 94.831) = 5.20, *p* = .013, η_p_^2^  = 0.08. To disentangle these effects, three‐way ANOVAs (Culture × View × AOI) were conducted separately for American Comics and Manga, allowing for a more detailed examination of the interaction patterns within each medium (Fig. 2). The results of ANOVAs are summarized in Table 2.

**Table 1:** Percentage of fixation duration for each AOI

	Objective page			Subjective page		
AOI	Focal object	Balloon	Background	Focal object	Balloon	Background
American comics						
American (*n* = 30)	37.44 (10.80)	34.14 (14.23)	28.42 (14.48)	29.16 (11.93)	35.59 (16.07)	35.25 (14.61)
Japanese (*n* = 34)	17.35 (5.15)	32.04 (15.54)	50.61 (13.25)	33.84 (7.58)	28.28 (14.41)	37.89 (11.35)
Manga						
American (*n* = 30)	35.00 (7.35)	34.27 (15.61)	30.73 (13.14)	29.06 (8.22)	41.88 (11.75)	29.06 (11.13)
Japanese (*n* = 34)	19.37 (4.68)	43.29 (17.71)	37.34 (15.58)	36.97 (10.19)	32.57 (14.54)	30.46 (15.12)

*Note*. Standard deviation in parentheses.

**Table 2:** Summary of the three‐way ANOVA results (Culture × View × AOI) for American comics and manga

	American comic			Manga		
Source	*F*	*p*	η_p_^2^	*F*	*p*	η_p_^2^
Culture	*F*(1, 62) = 0.74	.395	0.01	*F*(1, 62) = 7.05	.010	0.1
View	*F*(1, 62) = 9.23	.003	0.13	*F*(1, 62) = 5.11	.027	0.08
AOI	*F*(1.45, 89.81) = 6.91	.004	0.10	*F*(1.39, 85.92) = 6.04	.009	0.09
Culture × View	*F*(1, 62) = 18.76	< .001	0.23	*F*(1, 62) = 5.00	.029	0.08
Culture × AOI	*F*(1.45, 89.81) = 10.58	< .001	0.15	*F*(1.39, 85.92) = 1.25	.280	0.02
View × AOI	*F*(2, 124) = 3.23	.043	0.05	*F*(1.60, 98.95) = 7.84	.002	0.11
Culture × View × AOI	*F*(2, 124) = 29.69	< .001	0.32	*F*(1.60, 98.95) = 32.57	< .001	0.34

For American comics, the main effects of View and AOI were significant. The interactions of Culture × View, Culture × AOI, View × AOI, and Culture × View × AOI were also significant. A post‐hoc analysis revealed that the Culture × AOI interaction was significant for objective materials, *F*(1.49, 92.50) = 29.19, *p* < .001, η_p_^2^  = 0.32, but not for subjective materials, *F*(1.67, 103.34) = 2.53, *p* = .095, η_p_^2^  = 0.04. For objective materials, Americans fixated longer on focal objects than Japanese, *t*(62) = 9.21, *p* < .001, Cohen's *d* = 2.31, while Japanese fixated longer on the background than Americans, *t*(62) = −6.35, *p* < .001, Cohen's *d* = −1.59.

For manga, the main effects of Culture, View, and AOI, as well as the interactions of Culture × View, View × AOI, and Culture × View × AOI, were significant. A post‐hoc analysis revealed that the Culture × AOI interaction was significant for both objective materials, *F*(1.19, 73.47) = 10.70, *p* < .001, η_p_^2^  = 0.15, and subjective materials, *F*(1.73, 107.28) = 5.40, *p* = .008, η_p_^2^  = 0.08. For objective materials, Americans fixated longer on focal objects than Japanese, *t*(62) = 10.06, *p* < .001, Cohen's *d* = 2.52, while Japanese fixated longer on balloons than Americans, *t*(62) = −2.19, *p* = .032, Cohen's *d* = −0.55. For subjective materials, Japanese fixated longer on focal objects than Americans, *t*(62) = −3.32, *p* = .002, Cohen's *d* = −0.83, while Americans fixated longer on balloons than Japanese, *t*(62) = 2.76, *p* = .004, Cohen's *d* = 0.69.

As an additional analysis, we examined whether the continuous subjectivity ratings of each comic page obtained from the pretest predicted fixation on focal objects, and whether this relationship differed by culture. Separate linear mixed‐effects models were conducted for manga and American comics, with subjectivity rating (z‐scored), culture, and their interaction as fixed effects, and random intercepts for participants and pages. For American comics, subjectivity significantly predicted fixation on focal objects, *β* = .018, *SE* = .002, *t*(58.18) = 9.65, *p* < .001, but the culture × subjectivity interaction was not significant. For manga, the model revealed a significant main effect of subjectivity, *β* = .013, *SE* = .002, *t*(55.34) = 6.46, *p* < .001, indicating that higher perceived subjectivity was associated with greater attention to focal objects. The interaction between subjectivity and culture was significant, *β* = –.014, *SE* = .003, *t*(1196) = –5.21, *p* < .001. The positive association between subjectivity and focal attention was stronger for Japanese participants than for American participants.

### 3.2 Cognitive styles and experiences of reading comics

The AHS score of Japanese participants (*M* = 120.91, *SD* = 10.49) was significantly higher than that of American participants (*M* = 109.60, *SD* = 13.81), *t*(62) = 3.71, *p* < .001, Cohen's *d* = 0.93. This observed difference aligns with previous research (Nisbett & Miyamoto, 2005; Nisbett et al., 2001), which demonstrated that East Asians exhibit a more holistic cognitive style than North Americans do.

Regarding their experience reading comics, Japanese participants (*M* = 4.91, *SD* = 1.71) reported more extensive experience reading manga than American participants (*M* = 2.43, *SD* = 1.70), *t*(62) = 5.81, *p* < .001, Cohen's *d* = 1.45, while the experience of reading American comics did not differ significantly between the two cultures (American *M* = 2.27, *SD* = 1.64, Japanese *M* = 1.97, *SD* = 1.11), *t*(62) = 0.85, *p* = .397, Cohen's *d* = 0.21.

### 3.3 The effects of cognitive styles and comic reading experiences on visual attention

A hierarchical multiple regression analysis was conducted separately for American comics and manga. The dependent variable was the arcsined proportion of fixation duration on the focal object when viewing the objective pages. In the first step, culture (Japan = 0, USA = 1) was entered to examine baseline cultural differences. In the second step, mean‐centered scores for AHS and experience reading American comics and manga were added to assess the additional explanatory power of individual‐level characteristics. All predictors had variance inflation factor (VIF) values below 2.1, indicating the absence of multicollinearity issues.

The results are summarized in Table 3. For American comics, culture significantly predicted attentional focus on focal objects, accounting for 57.1% (*F*(1, 62) = 84.767, *p* < .001). The addition of the AHS score and reading experience in Step 2 had no effect. For manga, culture significantly predicted attentional focus on focal objects, accounting for 61.4% (*F* (1, 62) = 101.17, *p* < .001). In Step 2, both the AHS score (*β* = −0.175, *p* = .044) and reading experience of manga (*β* = .224, *p* = .026) emerged as significant predictors. A lower AHS score and greater reading experience were associated with higher scores for attentional focus on focal objects.

**Table 3:** Multiple linear regression predicting fixation duration to focal objects in objective pages

	Predictors	*R* ^2^	∆*R* ^2^	*β*	*F*	*p*
American comics						
Step 1		.571	.571		84.767	<.001
	Culture (0 = JP, 1 = US)			0.760		
Step 2		.571	.000		21.921	<.001
	Culture (0 = JP, 1 = US)			0.703		<.001
	AHS Score			−0.121		*n.s*.
	Reading Experience of American Comics			−0.020		*n.s*.
	Reading Experience of Manga			−0.062		*n.s*.
Manga						
Step 1		.614	.614		101.172	<.001
	Culture (0 = JP, 1 = US)			0.787		
Step 2		.650	.036		30.190	<.001
	Culture (0 = JP, 1 = US)			0.848		<.001
	AHS Score			−0.175		.044
	Reading Experience of American Comics			−0.024		*n.s*.
	Reading Experience of Manga			0.224		.026

Additional regression analyses were conducted to examine predictors of visual attention on subjective pages (see Appendix C). For American comics, neither culture nor any individual differences significantly predicted attention to focal objects. In contrast, for manga, culture significantly predicted fixation on focal objects (*β* = –.389, *p* = .002, *R* ^2^ = .151), and reading experience of manga was also a significant predictor (*β* = .303, *p* = .013).

## 4 Discussion

The primary purpose of this study was to investigate the interaction between readers’ cultural backgrounds and viewpoints drawn in the pages of comics. As hypothesized, the fixation duration while reading pages presenting an objective viewpoint demonstrated cultural differences in both American comics and manga (Culture × View): American readers allocated a greater proportion of visual attention to focal objects than did Japanese readers. Conversely, Japanese readers demonstrated prolonged visual attention to the background compared with their American counterparts, although this was observed only when viewing American comic materials (Culture × View × AOI). These findings suggest that even when adopting an objective viewpoint encompassing both focal and background information, participants with an American cultural background focus more easily on focal objects, while participants with a Japanese cultural background are likely to allocate their attention to the background as well as focal elements.

These eye‐tracking results align with previous findings that individuals from North American cultures tend to focus on focal objects, whereas individuals from East Asian cultures allocate attention to both backgrounds and focal objects when viewing visual scenes (Chua et al., 2005; Masuda & Nisbett, 2001). However, the current study extends these findings by demonstrating that cultural differences in attentional focus depend on the nature of the material—objective versus subjective viewpoints. For objective materials, the expected cultural differences were observed: American readers spent more time attending to focal objects, whereas Japanese readers allocated relatively more attention to backgrounds. By contrast, for subjective materials, these cultural differences diminished or even reversed. For manga's subjective pages, Japanese readers demonstrated greater attention to focal objects than Americans did, suggesting that the subjective perspective embedded in manga shifts Japanese readers toward a focal‐object‐oriented attentional style. This may reflect the influence of familiarity with the medium and implicit guidance provided by the artist. The results indicate that the attentional guidance embedded in the artist's design minimizes cultural differences for subjective materials, further supporting the hypothesis that comic artists simulate readers’ attention and guide their gaze (Cohn et al., 2012; McCloud, 1993).

To further investigate this pattern, we conducted additional analyses using the continuous subjectivity ratings of each page obtained from the pretest. The results showed that higher subjectivity ratings were associated with increased fixation on focal objects, and that this relationship was stronger among Japanese participants for manga. These findings suggest that subjective materials may shift Japanese readers’ attention toward a more object‐focused style, thus attenuating default cross‐cultural differences. These results illustrate that artistic design plays a powerful role in shaping visual cognition. However, this influence does not solely stem from artists’ attempts to accommodate viewers’ attentional tendencies. Rather, as Cohn (2024) emphasizes, both artists and readers share fluency in culturally shaped visual language systems. Artists create works using conventionalized narrative patterns that they themselves have internalized, and readers interpret them through similar experiential frameworks. Thus, attentional guidance embedded in comics reflects a shared cultural and cognitive system.

In addition to focal and background areas, we also observed cultural differences in attention to text balloons, particularly in manga pages. One possible explanation for this pattern lies in the content and communicative function of the text. Prior studies have shown that Americans tend to focus on explicit verbal content, while Japanese individuals may be more sensitive to emotional tone and implicit cues (e.g., Ishii, Reyes, & Kitayama, 2003). Although we did not code the linguistic content of the text balloons, future studies could examine whether such functional differences in text modulate attention across cultures.

The association between comic reading experience and reading processes aligns with the findings of previous studies. For example, Japanese children with extensive manga reading experience demonstrate more fluid eye movements that align with manga frame layouts than children with limited experience (Nakazawa, 2002). Additionally, frequent manga reading during childhood predicts visual narrative comprehension ability (Cohn & Kutas, 2017). These findings, combined with the results of this study, suggest that extensive manga reading experience enables individuals to align their eye movements and reading patterns with those intended by the comic artists. Given that manga predominantly employs a subjective viewpoint, readers with extensive manga reading experience may develop expertise in focusing on focal objects. Conversely, American comics primarily utilize an objective viewpoint; therefore, reading experience with this medium may not induce attentional bias toward focal objects. Notably, because reading experience with American comics varied minimally among participants, its predictive power for attentional allocation may have been constrained. Thus, the lack of an observed effect could reflect distributional limitations rather than a true absence of influence.

Furthermore, the results demonstrated that individuals with a more holistic orientation exhibited a reduced focus on focal objects, although this effect was exclusively observed in manga reading. This qualified association between holistic scores and attention suggests that conceptual cognitive styles, as measured by self‐report scales, are reflected in overt visual attention during some comic reading. However, the absence of a significant association in American comics limits this generalization. Mixed evidence from cross‐cultural psychology suggests that self‐report measures may not always align with behavioral processes (Heine, Lehman, Peng, & Greenholtz, 2002; Oyserman, Coon, & Kemmelmeier, 2002; Shimizu & Uleman, 2021). A systematic investigation is necessary to identify the conditions under which this association can be observed.

This study provides initial empirical evidence that readers’ cultural backgrounds interact with comic artists’ viewpoint choices to guide visual attention. By demonstrating that cultural differences in attentional styles, well‐documented within the framework of cross‐cultural psychology, also manifest in patterns of visual attention when engaging with visual narratives such as comics, these findings extend prior research into new contexts. Specifically, the results highlight how artistic designs, including subjective and objective viewpoints, shape visual processing in ways that both reflect and override culturally ingrained attentional tendencies. Additionally, our results underscore the dual influence of culture and medium‐specific fluency. While culture shapes a general attentional style, familiarity with manga conventions can reinforce or override these tendencies, especially in subjective pages. This interaction shows that cultural differences and domain‐specific expertise are not mutually exclusive but rather complementary in guiding readers’ visual attention. Consequently, this study enhances our understanding of cross‐cultural differences in visual processing and comic reading behaviors, offering new insights into the intricate interplay between culture, cognition, and visual narratives.

However, this study has some limitations. First, the participants were presented with comic pages individually, which deviated from typical comic reading practices. To capture the process of reading comics as a visual narrative accurately, it is necessary to investigate the reading of sequential pages. Second, as this study employed authentic comics, it was not feasible to precisely determine the author's intentions regarding the guidance of readers’ visual attention on each page. Future research should conduct more systematic investigations into the interactions between authorial intentions and reader responses. Finally, while our current study assessed subjectivity based on readers’ phenomenological impressions, future research may complement these findings by systematically coding structural elements of the stimuli. For instance, applying formal annotation schemes to quantify background density, point‐of‐view shifts, or focal framing (Atilla et al., 2023; Cohn, 2024) could help clarify how such visual narrative features correspond to perceived subjectivity and shape visual attention.

## Conflict of interest

We declare that there are no competing financial or nonfinancial interests related to this work.

## Funding

This study was supported by a Grant‐in‐Aid for Japanese Scientific Research KAKENHI to Yuki Shimizu (No. 17K04308 and No. 21H00939) and Motohiro Kozawa (No. 19H01664).

## Data Availability

The data analyzed in this study are available on the Open Science Framework: https://osf.io/7jvrk/?view_only=d66f4f9915c6443eaab7dff1326f0fe0

## Appendix A

### Source of stimulus comic pages

**Table:**

Comic No.	Comic type	Viewpoint	Title	Publisher	Author	Volume	Page
1	American comics	objective	Aquaman Vol. 1: The Trench	DC Comics	Geoff Johns, Ivan Reis	1	65
2	American comics	objective	Aquaman Vol. 1: The Trench	DC Comics	Geoff Johns, Ivan Reis	1	123
3	American comics	objective	Batman: The Long Halloween	DC Comics	Jeph Loeb	1	53
4	American comics	objective	Batman: The Long Halloween	DC Comics	Jeph Loeb	1	232
5	American comics	objective	Cosmic Odyssey: The Deluxe Edition	DC Comics	Jim Starlin	1	8
6	American comics	objective	Cosmic Odyssey: The Deluxe Edition	DC Comics	Jim Starlin	1	30
7	American comics	objective	Cosmic Odyssey: The Deluxe Edition	DC Comics	Jim Starlin	1	67
8	American comics	objective	Cosmic Odyssey: The Deluxe Edition	DC Comics	Jim Starlin	1	132
9	American comics	objective	Spider‐Man: Homecoming Prelude	Marvel Comics	Will Pilgrim	1	50
10	American comics	objective	Wolverine: Old Man Logan	Marvel Comics	Mark Millar	1	198
11	American comics	subjective	Aquaman Vol. 1: The Trench	DC Comics	Geoff Johns, Ivan Reis	1	56
12	American comics	subjective	Aquaman Vol. 1: The Trench	DC Comics	Geoff Johns, Ivan Reis	1	118
13	American comics	subjective	Batman: The Long Halloween	DC Comics	Jeph Loeb	1	131
14	American comics	subjective	Batman: The Long Halloween	DC Comics	Jeph Loeb	1	141
15	American comics	subjective	Batman: The Long Halloween	DC Comics	Jeph Loeb	1	233
16	American comics	subjective	Batman: The Long Halloween	DC Comics	Jeph Loeb	1	312
17	American comics	subjective	Cosmic Odyssey: The Deluxe Edition	DC Comics	Jim Starlin	1	182
18	American comics	subjective	Spider‐Man: Homecoming Prelude	Marvel Comics	Will Pilgrim	1	32
19	American comics	subjective	Spider‐Man: Homecoming Prelude	Marvel Comics	Will Pilgrim	1	62
20	American comics	subjective	Wolverine: Old Man Logan	Marvel Comics	Mark Millar	1	98
21	Manga	objective	BLACK CAT	SHUEISHA	Kentaro Yabuki	1	104
22	Manga	objective	BLACK CAT	SHUEISHA	Kentaro Yabuki	1	127
23	Manga	objective	BLEACH	SHUEISHA	Taito Kubo	1	10
24	Manga	objective	BLEACH	SHUEISHA	Taito Kubo	1	69
25	Manga	objective	NARUTO	SHUEISHA	Masashi Kishimoto	1	153
26	Manga	objective	Corpse Princess	Square Enix	Yoshiichi Akahito	1	31
27	Manga	objective	Black clover	SHUEISHA	Yuki Tabata	1	92
28	Manga	objective	Black clover	SHUEISHA	Yuki Tabata	1	117
29	Manga	objective	My hero academia	SHUEISHA	Kohei Horikoshi	1	97
30	Manga	objective	My hero academia	SHUEISHA	Kohei Horikoshi	1	184
31	Manga	subjective	BLACK CAT	SHUEISHA	Taito Kubo	1	197
32	Manga	subjective	NARUTO	SHUEISHA	Masashi Kishimoto	1	44
33	Manga	subjective	Corpse Princess	Square Enix	Yoshiichi Akahito	1	14
34	Manga	subjective	Corpse Princess	Square Enix	Yoshiichi Akahito	1	18
35	Manga	subjective	Corpse Princess	Square Enix	Yoshiichi Akahito	1	23
36	Manga	subjective	Corpse Princess	Square Enix	Yoshiichi Akahito	1	151
37	Manga	subjective	Corpse Princess	SHUEISHA	Yoshiichi Akahito	1	157
38	Manga	subjective	Black clover	SHUEISHA	Yuki Tabata	1	129
39	Manga	subjective	My hero academia	SHUEISHA	Kohei Horikoshi	1	28
40	Manga	subjective	Future Diary	SHUEISHA	Sakae Esuno	1	36

## Appendix B

### Details on the pretest for selecting stimuli

**Table B1:** Means and standard deviations of pretest viewpoint ratings for each comic page

Comic No.	*Mean*	*SD*
1	3.12	1.66
2	2.82	1.79
3	3.24	1.90
4	3.50	2.13
5	3.41	1.85
6	3.41	1.97
7	2.85	1.83
8	2.56	1.74
9	2.59	1.77
10	2.79	1.62
11	5.12	1.59
12	4.56	1.68
13	4.50	1.74
14	4.53	1.56
15	4.50	1.74
16	4.71	1.92
17	4.59	1.72
18	4.35	1.88
19	4.43	1.61
20	4.56	1.65
21	3.44	1.90
22	3.47	1.74
23	3.03	1.79
24	3.15	1.37
25	3.38	1.78
26	3.47	1.40
27	3.26	1.79
28	3.33	1.77
29	3.41	1.80
30	3.50	1.65
31	4.62	1.70
32	4.50	1.58
33	4.53	1.61
34	4.59	1.66
35	4.71	1.49
36	4.56	1.61
37	4.65	1.61
38	4.59	1.57
39	4.50	1.77
40	5.03	1.48

Initially, six American comic books and seven Japanese manga publications were selected, all of which were available in both Japanese and English languages. Two artists and a psychologist (all of whom are included among the authors) listed up 30 pages of American comics and 30 pages of manga that were deemed to employ subjective or objective viewpoints. These pages were subjected to preliminary testing by 16 American and 18 Japanese undergraduate and graduate students to validate the perspective that is considered to be adopted. First, pretest participants were provided with the definitions of objective and subjective viewpoints (objective viewpoint: “The viewpoint which views characters and situations in the setting from a distant point”; subjective viewpoint: “The viewpoint which makes the reader feel as if they perceive through their own viewpoint in the story”). Then, they were presented with each page and asked to indicate the extent to which they perceived an objective or subjective viewpoint to be employed on each page, using a 7‐point scale (1 = objective to 7 = subjective). After excluding pages containing three or fewer panels, we selected 10 subjective pages from each American comic and manga with an average score above 4.5 and 10 objective pages from each American comic and manga with an average score below 3.5. The mean and standard deviation of ratings for each comic are presented in Table B1.

## Appendix C

### Multiple linear regression predicting fixation duration to focal objects in subjective pages

**Table:**

	Predictors	*R* ^2^	∆*R* ^2^	*β*	*F*	*p*
American comics						
Step 1		.044	.044		2.858	*n.s*.
	Culture (0 = JP, 1 = US)			−0.210		
Step 2		.068	.024		0.498	*n.s*.
	Culture (0 = JP, 1 = US)			0.137		*n.s*.
	AHS Score			−0.136		*n.s*.
	Reading Experience of American Comics			−0.030		*n.s*.
	Reading Experience of Manga			0.115		*n.s*.
Manga						
Step 1		.151	.151		11.029	.002
	Culture (0 = JP, 1 = US)			−0.389		
Step 2		.260	.210		5.183	.001
	Culture (0 = JP, 1 = US)			−0.389		<.001
	AHS Score			−0.022		*n.s*.
	Reading Experience of American Comics			0.072		*n.s*.
	Reading Experience of Manga			0.303		.013
